# Personalized positive-end expiratory pressure using electrical impedance tomography in ARDS patients: a systematic review and meta-analysis

**DOI:** 10.1016/j.aicoj.2026.100049

**Published:** 2026-03-16

**Authors:** Michela Rauseo, Danila Azzolina, Gaetano Scaramuzzo, Mohd Rashid Khan, Paolo Vetuschi, Francesco Paolo Padovano, Antonello Discenza, Lucia Distaso, Lucia Mirabella, Antonella Cotoia, Savino Spadaro, Gilda Cinnella

**Affiliations:** a University of Foggia, Department of Medical and Surgical Science, Anesthesia and Intensive Care Medicine, Policlinico Riuniti di Foggia, Italy; b Biostatistics and Clinical Trial Methodology Unit, Clinical Research Center DEMeTra, Department of Translational Medical Science, University of Naples Federico II, Naples, Italy; c Department of Translational Medicine, University of Ferrara, Saint’ Anna Hospital, Ferrara, Italy; d Department of Cardiac, Thoracic, Vascular Sciences and Public Health, University of Padua, Padua, Italy; e Department of Cardiac Anesthesia, Policlinico Riuniti di Foggia, Italy; f Azienda Ospedaliera Universitaria di Ferrara, Arcispedale San’ Anna, Ferrara, Italy

**Keywords:** Electrical impedance tomography, ARDS, PEEP titration, Oxygenation, Respiratory compliance, Lung mechanics, Mechanical ventilation, Driving pressure, Mechanical power.

## Abstract

**Objective:**

Personalized mechanical ventilation strategies can improve outcomes in patients with Acute Respiratory Distress Syndrome (ARDS). However, the optimal method for titrating positive end-expiratory pressure (PEEP) remains controversial. Electrical Impedance Tomography (EIT), by enabling real-time, regional assessment of lung regional characteristics, offers potential advantages to guiding PEEP titration as compared to conventional methods.

**Data sources and study selection:**

We conducted a systematic review and meta-analysis of randomized clinical trials and observational studies comparing EIT-guided versus conventional PEEP titration in adult ARDS. The review was conducted according to the PRISMA 2020 guidelines. Moreover, the review was conducted in accordance with the MOOSE (Meta-analysis of Observational Studies in Epidemiology) recommendations, where applicable, and with the registered protocol (PROSPERO ID: CRD420251015187). The primary outcomes were the PaO_2_/FiO_2_ ratio and respiratory system compliance (Crs). Secondary outcomes included driving pressure (ΔP), mechanical power (MP), and mortality. Random-effects meta-analysis was performed. Risk of bias and GRADE assessments were conducted using the ROB 2.0 and ROBINS-I tools, depending on the study design.

**Data extraction and data synthesis:**

Nine studies (n = 356 patients) were included. EIT-guided PEEP titration was associated with an improvement in oxygenation, expressed as PaO_2_/FiO_2_ ratio, in the EIT guided PEEP group (MD + 60.81; 95% CI 30.37–91.25), with low heterogeneity. Furthermore, significant improvement in respiratory system compliance was observed in EIT guided PEEP (MD + 6.81 mL/cm H_2_O; 95% CI 3.73–9.89). No statistically significant difference was observed in driving pressure between groups (MD −0.78 cm H_2_O; 95% CI −1.63 to 0.07). Mechanical power showed a non-significant difference across groups (MD −0.76 J/min; 95% CI −2.30 to 0.78). Mortality did not differ between groups (risk ratio 0.88; 95% CI 0.45–1.72).

**Conclusion:**

EIT-guided PEEP titration improves oxygenation and respiratory system compliance in patients with ARDS, supporting its role as a physiology-based strategy for ventilatory personalization. Further evidence are needed to determine whether these physiological improvements translate into better clinical outcomes.

## Highlights

• EIT-guided PEEP titration improves oxygenation in patients with ARDS.
• Individualized PEEP adjustment using EIT increases respiratory system compliance.
• Electrical impedance tomography allows bedside, real-time assessment of lung mechanics.
• EIT-guided ventilation supports physiologically individualized PEEP optimization in ARDS.

## Introduction

Acute Respiratory Distress Syndrome (ARDS) remains a life-threatening condition characterized by severe hypoxemia, heterogeneous lung impairment, and poor outcomes despite lung-protective ventilation strategies [1,2]. Optimal titration of positive end-expiratory pressure (PEEP) is a cornerstone of ARDS management, aiming at preventing alveolar collapse while minimizing overdistension [3]. However, selecting the “best PEEP” remains a clinical challenge due to the heterogeneity in lung recruitability and respiratory mechanics [4].

Traditional approaches to PEEP titration, such as PEEP/FiO_2_ tables or compliance-based adjustments, often lack regional specificity and may fail to capture heterogeneous regional lung behavior [5,6]. Electrical Impedance Tomography (EIT) is a noninvasive bedside imaging tool that provides dynamic, real-time information on lung ventilation distribution. EIT-guided PEEP titration supports individualized ventilator management by providing information on regional ventilation patterns, while emphasizing that PEEP determination depends on a global evaluation [7,8].

Growing evidence have suggested that EIT-guided PEEP titration may improve gas exchange and lung mechanics, but its impact on clinical and physiological outcomes remains unclear [9]. Previous meta-analysis reported some benefits but included studies with heterogeneous designs [10] and methodological limitations, such as small sample size, variable definitions of ARDS, and inconsistent PEEP titration protocols [11,12]. Moreover, the certainty of the evidence was not formally evaluated, as no GRADE assessment was conducted. Furthermore, few studies have characterized patient outcomes based on ARDS severity or patient characteristics (e.g., obesity, COPD, post-cardiac surgery). In addition, few studies have explored how EIT-guided titration affects mechanical power or energy distribution [10].

The COVID-19 pandemic has underlined the need for personalized approaches to mechanical ventilation. The heterogeneity of ARDS phenotypes observed during and after the pandemic has renewed interest in bedside monitoring to guide individualized treatment strategies. In this context, EIT has gained attention as a promising tool in research and real-world clinical decision-making. However, integrating EIT in PEEP titration protocols still varies widely across centers, and robust evidence is needed to support its broader clinical implementation [[13], [14], [15]] especially in ARDS populations with substantial patient heterogeneity.

This systematic review and meta-analysis was conducted to compare the effects of EIT-guided PEEP titration with conventional PEEP approaches in adults ARDS patients. We evaluated physiological outcomes (oxygenation, compliance, driving pressure, mechanical power) and short-term mortality, focusing on methodological robustness ensured through strict inclusion criteria, risk-of-bias assessment, and GRADE evaluation, and on the clinical relevance of outcomes.

## Materials and methods

This systematic review and meta-analysis followed the PRISMA 2020 guidelines. Moreover, the review was conducted in alignment with the MOOSE (Meta-analysis Of Observational Studies in Epidemiology) recommendations, where applicable, and in accordance with the registered protocol. The review protocol was prospectively registered in PROSPERO (ID: CRD420251015187)

We searched PubMed/MEDLINE, Embase, and Web of science from January 1, 2012, to February 28, 2025, using the following keywords: “Electrical Impedance Tomography” OR “EIT” AND “ARDS” OR “acute respiratory distress syndrome” AND “PEEP” OR “positive end-expiratory pressure” OR “ventilation” OR “mechanical ventilation”. Only English-language studies were included.

The whole search strategy is available in the Supplementary Material. We included trials and observational studies comparing EIT-guided PEEP titration with conventional PEEP strategies in adult ARDS patients. Study screening (titles/abstracts and full texts), data extraction, and risk-of-bias assessments were all conducted independently and in duplicate by two reviewers. Any discrepancies were resolved through discussion, and when consensus was not immediately achieved, a third senior reviewer was consulted for adjudication. Trial registries and grey literature were not systematically searched.

**Fig. 1:**
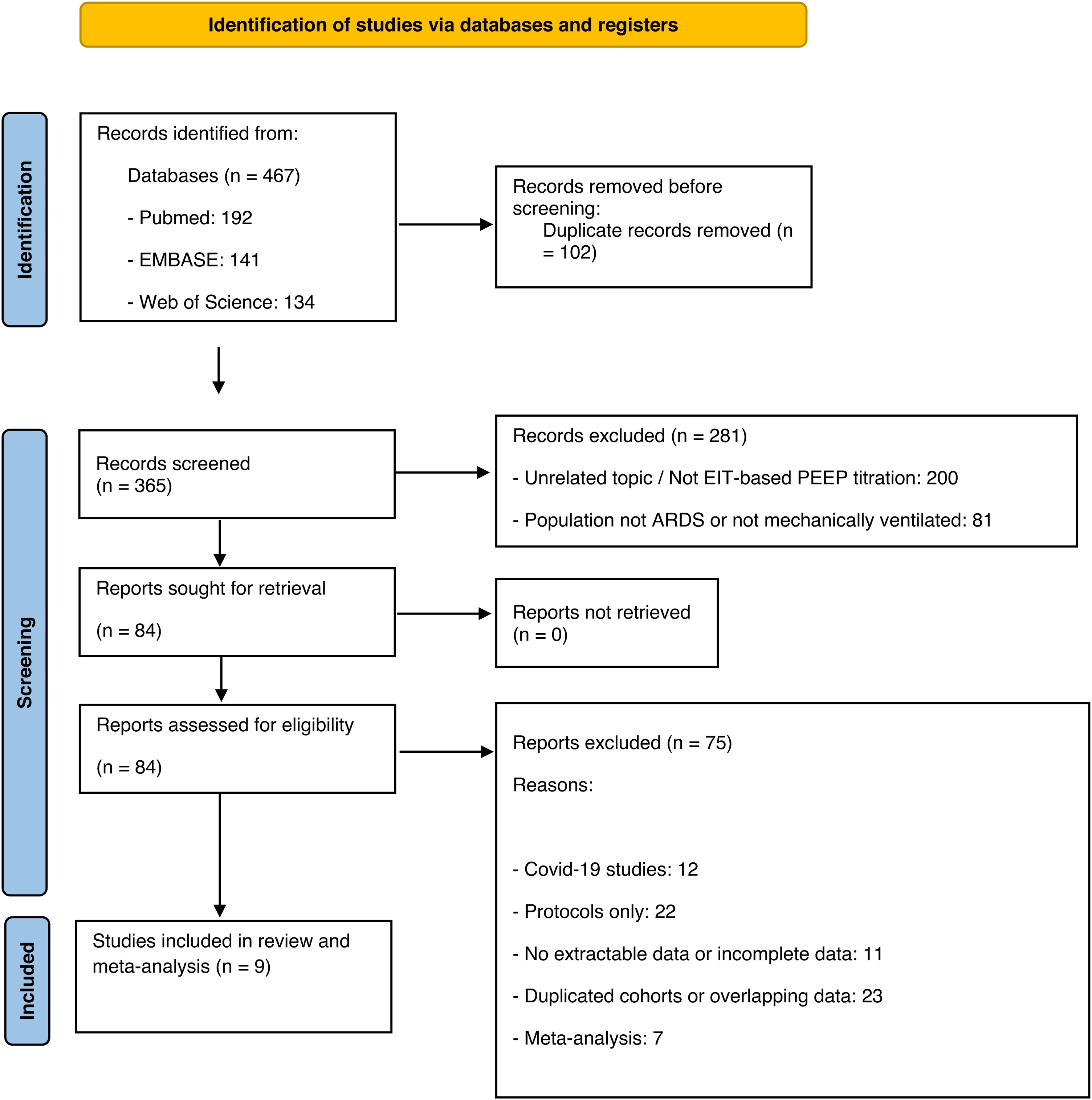
PRISMA 2020 Flow diagram illustrating the identification, screening, eligibility assessment, and inclusion of studies in the systematic review and meta-analysis.

We excluded studies involving COVID-19 patients, pediatric populations, case reports, simulation studies, and trials using PEEP titration based solely on pressure/volume (P/V) curves, driving pressure (ΔP), or oxygenation without EIT guidance. Two reviewers (MR, SS) screened all titles and abstracts (Table 1). Disagreements were resolved by consensus with a third reviewer (DA), and all selection was documented in a PRISMA flow diagram (Fig. 1).

**Table 1:** Summary of included studies and key findings.

Study	Design	Sample Size	Setting	Outcomes Measured	Conventional- PEEP	EIT-guided PEEP titration	Key Findings
Jimenez 2023	RCT	12	ICU	PaO_2_/FiO_2_, ΔP, MP, compliance, mortality	High PEEP/FiO_2_ table	Decremental PEEP Trial (Drop ΔEELI 10%)	Lower PEEP level, improved compliance, reduced ΔP, reduced MP with EIT-guided strategies
He 2021	RCT	117	ICU	Mortality, Ventilator-free days, ICU-LOS, SOFA Day 1−2	Low PEEP/FiO_2_ table	EIT Ventilation distribution	No difference in PEEP level, mortality, ΔP or compliance. SOFA score reduced in EIT guided strategies
Zhao 2019	RCT	55	ICU	PaO_2_/FiO_2_, ΔP, compliance, mortality	Static Pressure/Volume curve (2 cmH_2_O above LIP)	Intercept point cumulated collapse/overdistension curve	Higher PEEP level, improved PaO_2_/FiO_2_, ΔP and compliance with EIT-guided PEEP
Hsu 2021	RCT	87	ICU	PaO_2_/FiO_2_, ΔP, compliance, mortality	Static Pressure/Volume curve (maximal hysteresis)	Intercept point cumulated collapse/overdistension curve	Higher PEEP set in the conventional group. Better compliance and oxygenation, lower mortality with EIT-guided PEEP
Scaramuzzo 2020	Observational	20	ICU	Distribution of ventilation and transpulmonary- ΔP	PEEP/FiO_2_ table	Incremental PEEP trial, Silent spaces total ≤15%	No difference in PEEP level. Increased driving pressure and reduced silent spaces with EIT-guided PEEP
Becher 2021	Observational	20	ICU	PaO_2_/FiO_2_, compliance, stress and strain	Low PEEP/FiO_2_ table	A sustained-inflation maneuver with Paw of 40 mbar applied for a duration of 40 and regional Compliance	Higher PEEP level, improved oxygenation, no change in compliance, and higher stress and strain with EIT-guided PEEP
Liu 2022	Observational	27	ICU	PaO_2_/FiO_2_, ΔP, MP, compliance, GI index, mortality	Low PEEP/FiO_2_ table	Incremental PEEP trial; minimum GI index value	Lower PEEP level, reduction in MP, Ppeak and Pplat with EIT-guided PEEP
Eronia 2017	Observational	16	ICU	PaO_2_/FiO_2_, ΔP, MP, compliance	Low PEEP/FiO_2_ table	Application of a RM, with a positive pressure of 40 cmH2O for 40 s; PEEP EIT based on EELI variation	Higher PEEP levels with EIT-based method, improved oxygenation, reduced ΔP.
Cinnella 2015	Observational	15	ICU	PaO_2_/FiO_2_, ΔP, elastance	PEEP/FiO2 table	Decremental PEEP trial (ROI ventral/dorsal; Vt ventral/dorsal)	Higher PEEP level with EIT-based method. Improved oxygenation, decreased ΔP

Overview of the included studies reporting design, sample size, setting, measured outcomes, and main results comparing EIT-guided PEEP and conventional PEEP.

Abbreviations: ΔP, Driving Pressure; MP, Mechanical Power; PEEP, Positive End-Expiratory Pressure; ARDS, Acute Respiratory Distress Syndrome; ICU, Intensive Care Unit; EIT, Electrical Impedance Tomography.

### Data extraction and outcomes

We described the studies considering the study design, patients’ characteristics, type of intervention (EIT-guided PEEP vs. conventional PEEP), and the following outcomes:

• Primary outcomes: PaO_2_/FiO_2_ ratio and respiratory system compliance (Crs);
• Secondary outcomes: ΔP, mechanical power (MP), and mortality.

For each group, we collected means and standard deviations (SD) for physiological variables.

### Risk of bias and quality assessment

Risk of bias was assessed using the RoB 2.0 tool for RCTs and ROBINS-I for observational studies. For RoB 2 The overall risk-of-bias judgment was derived according to the RoB 2 algorithm, whereby a study was classified as low risk only if all domains were rated as low; the presence of at least one domain with some concerns resulted in an overall rating of some concerns; and at least one high-risk domain resulted in an overall high risk of bias. For ROBINS-I The overall risk-of-bias judgment corresponded to the highest level of bias identified across domains, in accordance with ROBINS-I guidance [16,17]. The certainty of evidence was rated using the GRADE approach across all outcomes (risk of bias, inconsistency, indirectness, imprecision, and publication bias) [18]. Two reviewers independently applied the GRADE criteria using GRADEpro GDT software [19].

For each outcome, the certainty of evidence was assessed independently across the five GRADE domains (risk of bias, inconsistency, indirectness, imprecision, and publication bias).

Risks of bias judgments were driven by the methodological quality of randomized trials, while observational studies were considered supportive but not dominant contributors when RCT data were available. Inconsistency was evaluated using both statistical heterogeneity (I²) and qualitative assessment of effect direction. Indirectness was examined considering population, intervention heterogeneity (different EIT titration protocols), and the physiological nature of outcomes. Imprecision was judged based on total sample size, width of confidence intervals, and whether effect estimates crossed clinically meaningful thresholds. Publication bias was assessed exploratory explored using a funnel plot inspection.

Discrepancies were resolved through discussion or consultation with a third reviewer. For each outcome, the overall certainty of evidence was categorized as high, moderate, low, or very low, based on the collective evaluation of these domains. GRADE Summary of Findings tables were compiled for all primary and secondary outcomes (Table 2).

**Table 2:** GRADE summary of findings.

Outcome	No. of Studies	Certainty (Revised)	Effect (95% CI)	Interpretation
PaO_2_/FiO_2_*	9	Moderate	+60.81 [30.37, 91.25]	EIT-guided PEEP improves oxygenation; evidence consistent but based largely on short-term physiologic endpoints and mixed study designs.
Compliance (Crs)*	9	Moderate	+6.81 [3.73, 9.89]	Improved respiratory system compliance with EIT; consistent effect, but indirect (physiologic surrogate) and partly observational evidence.
Driving Pressure (ΔP)*	9	Moderate	−0.78 [−1.63, 0.07]	Trend toward reduction; confidence interval crosses null; consistent direction but imprecision limits certainty.
Mechanical Power (MP)*	4	Low–Moderate	−0.76 [−2.30, 0.78]	No significant difference; limited number of studies and imprecision reduce certainty.
Mortality**	4	Low	RR 0.88 [0.45, 1.72]	No clear mortality effect; underpowered analysis with wide confidence interval and moderate heterogeneity.

Summary of evidence quality and effect estimates for each outcome according to the GRADE framework, including certainty of evidence, direction of effect, and clinical interpretation.

Abbreviations: MP = Mechanical Power; PEEP = Positive End-Expiratory Pressure; ARDS = Acute Respiratory Distress Syndrome.

* Mean difference.

** Risk ratio.

### Statistical analysis

For each of the five outcomes - PaO_2_/FiO_2_ ratio, Crs, ΔP, MP, and mortality - we performed a quantitative synthesis using a random-effects model to account for potential heterogeneity across studies. Continuous outcomes were summarized using mean differences (MD) with corresponding 95% confidence intervals (CIs), while binary outcomes (mortality) were expressed as risk ratios (RRs) with 95% CIs.

The available evidence comprises both parallel-group and paired (within-patient) designs; for this reason we implemented a design-aware inverse-variance meta-analytic approach. Parallel studies were analyzed assuming independent groups, whereas paired studies incorporated a correlation-adjusted variance formulation. As within-subject correlation is not reported, we treated this parameter as an explicit assumption of 0.5, as indicated in other studies when information about correlation is not directly available [20].

For studies employing within-subject (paired) designs, effect sizes were calculated using a correlation-adjusted variance approach. When mean differences between EIT-guided and conventional PEEP were reported within the same patients, the standard error of the paired mean difference was derived using the formula:

$$Var(D)=SD_{1}^{2}+SD_{2}^{2}-2r(SD_{1}\times SD_{2})$$

where $SD_{1}$ and $SD_{2}$ represent the standard deviations under each condition, and r denotes the within-subject correlation between paired measurements.

Because primary studies did not report the correlation coefficient, we assumed a conservative mid-range value of r = 0.5, consistent with methodological guidance for meta-analyses of paired physiological data when empirical estimates are unavailable. To assess the robustness of this assumption, sensitivity analyses were performed using alternative plausible correlation values (r = 0.25 and r = 0.75). These analyses evaluated the impact of the assumed correlation on pooled variance estimates and overall effect sizes. Heterogeneity among included studies was assessed using the I² statistic, with values of 25%, 50%, and 75% interpreted as low, moderate, and high heterogeneity, respectively. A Cochran’s Q test was also performed to test for heterogeneity, with a p-value <0.10 considered significant due to its low power in meta-analyses with small sample sizes.

To explore potential sources of heterogeneity and assess the robustness of our findings, we conducted stratified analyses based on study design (RCTs vs observational studies), as illustrated in the forest plots. These stratifications allowed us to assess whether effect sizes differed systematically according to the study design.

Publication bias was evaluated visually through funnel plots for each outcome. Symmetry of the funnel plot was taken as an indication of the absence of small-study effects or publication bias.

All meta-analyses were performed using R (version 3.4.2) [21] using the metafor package [22]. Two-tailed p-values less than 0.05 were considered statistically significant for all effect estimates.

## Results

### Study selection and characteristics

The final analysis included 9 studies, including 356 adult patients with ARDS. Four of these were randomized controlled trials, and five were observational studies [9,[23], [24], [25], [26], [27], [28], [29], [30]]. All studies applied lung-protective ventilation strategies and compared EIT-guided PEEP titration with either PEEP/FiO_2_ tables, fixed PEEP levels, or compliance-based adjustments. Baseline PEEP values across the included studies ranged from 8 to 18 cm H_2_O in the EIT group and from 7 to 17 cm H_2_O in the conventional group. Outcome measurements were obtained immediately after PEEP titration or following short stabilization periods. Most patients were classified as moderate to severe ARDS.

COVID-19 studies were excluded (Table 1, Table S1). Full study selection is detailed in the PRISMA flowchart (Fig. 1).

The detailed Risk of bias domain-level assessments are presented in Tables S2 and S3. Among randomized trials, no study was judged at high risk of bias. However, all were classified as having some concerns overall according to the RoB 2 algorithm. This judgment was primarily driven by open-label designs, incomplete reporting of allocation concealment in parallel trials, and potential deviations inherent to crossover physiology studies. Importantly, measurement bias was considered low across trials, as the primary outcomes were objective physiological variables (e.g., oxygenation, compliance, driving pressure, mechanical power) and mortality.

In contrast, all non-randomized studies were judged at serious risk of bias under ROBINS-I. The principal source of bias was confounding related to before–after designs, sequential protocols, or the use of historical controls. Although intervention classification and outcome measurement were generally robust and objectively defined, the absence of randomization and the potential influence of time-dependent or co-intervention effects limited internal validity.

### Primary outcomes

#### PaO_2_/FiO_2_ ratio

**Fig. 2:**
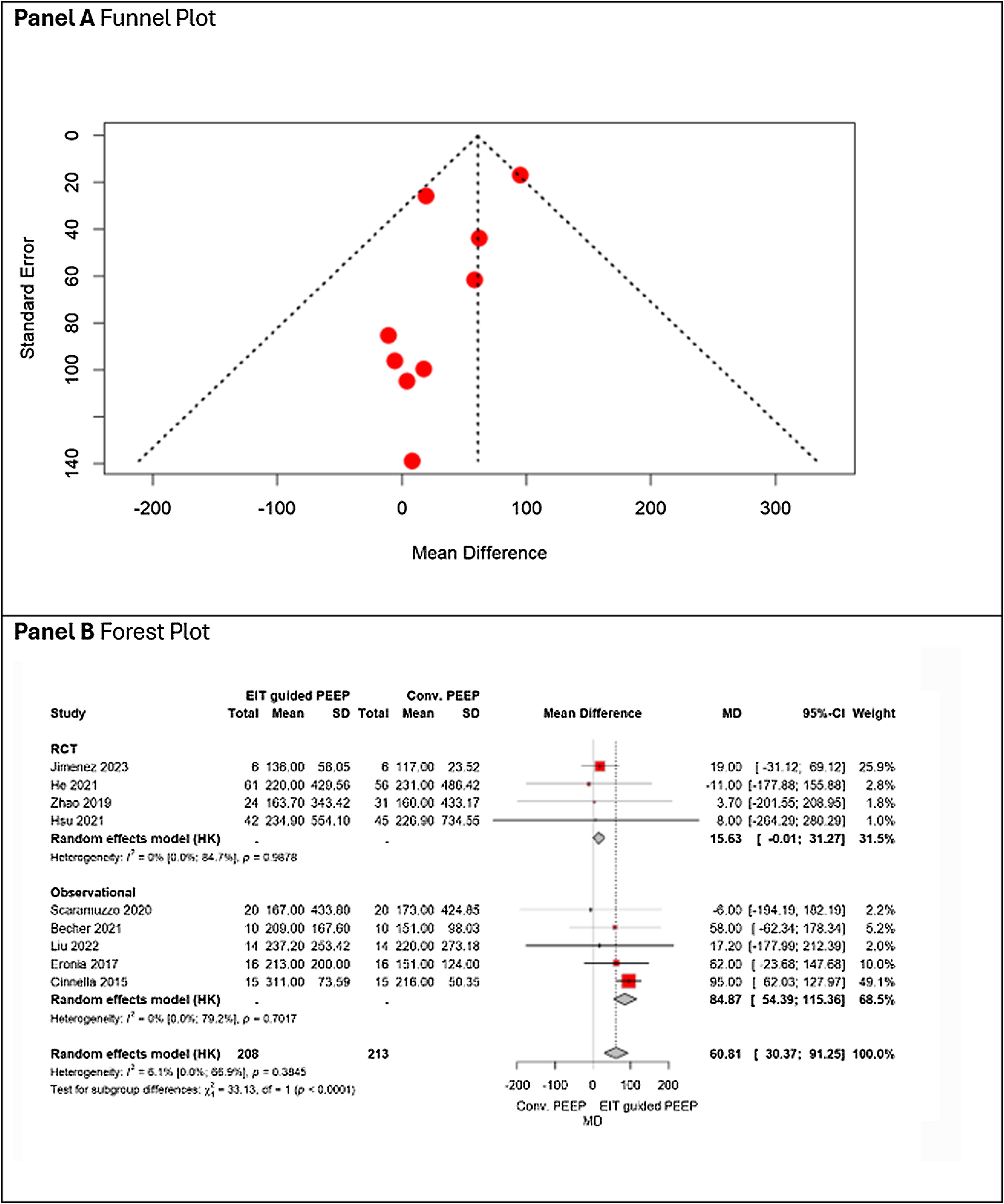
Panel A shows the funnel plot, and Panel B the forest plot, displaying the pooled effect estimates of EIT-guided PEEP versus conventional PEEP on PaO_2_/FiO_2_, stratified by study design (RCT vs observational).

Personalized PEEP guided by EIT was associated with a significant improvement in PaO_2_/FiO_2_ ratio compared with conventional approach (MD + 60.81, 95% CI 30.37–91.25), with low between-study heterogeneity (I² = 6.1%). Subgroup analyses suggested a stronger effect in observational studies in comparisons with RCTs (Fig. 2, Panel B). No evident asymmetry was observed on visual inspection. (Fig. 2, Panel A) and GRADE certainty of evidence was rated as moderate (Table 2). Sensitivity analyses showed overall consistency with the primary analysis. The only exception was driving pressure, which demonstrated a borderline effect in the main model and became marginally statistically significant when a within-subject correlation of 0.25 was assumed (Appendix 2).

#### Static respiratory system compliance

**Fig. 3:**
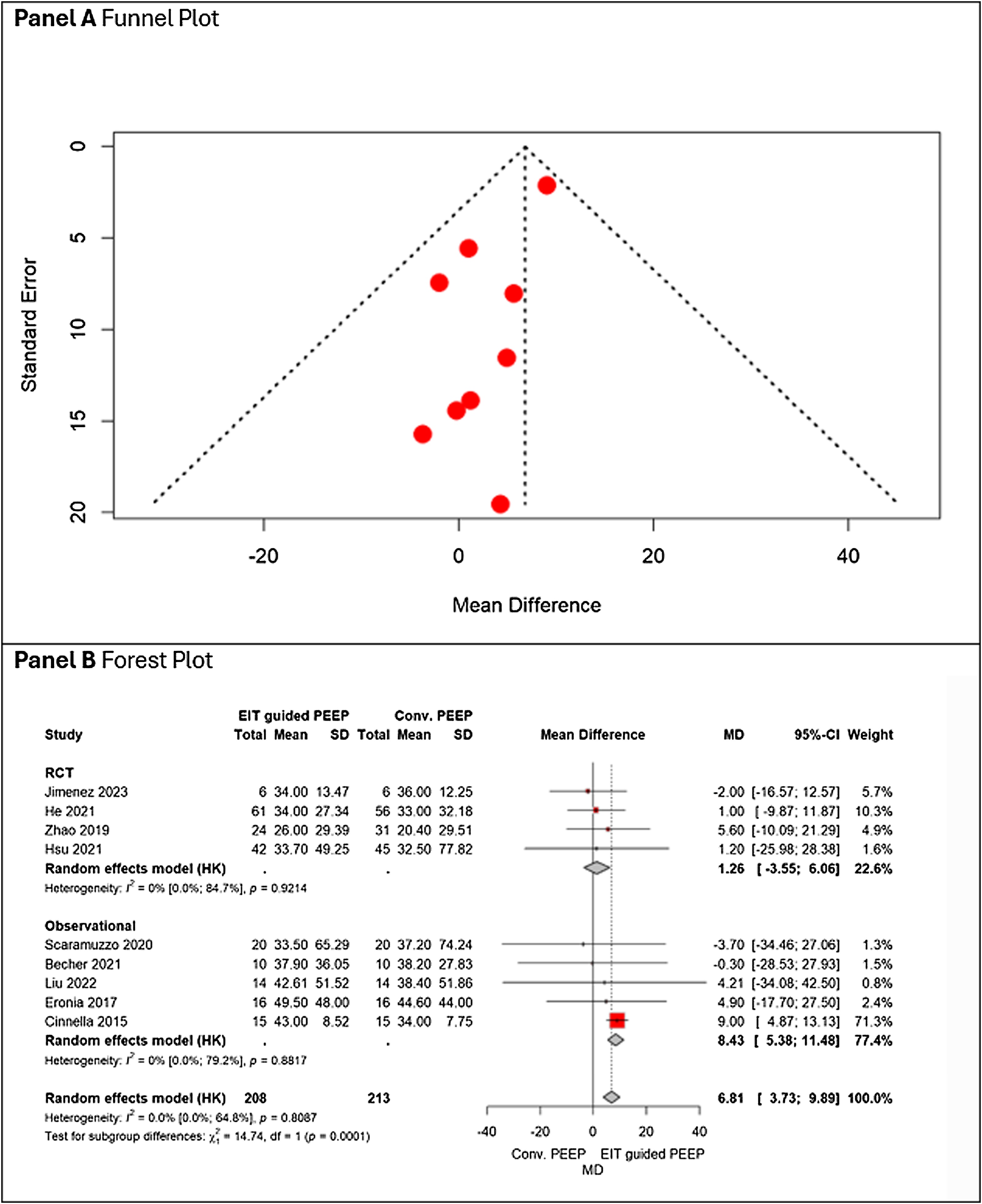
Panel A shows the funnel plot, and Panel B the forest plot, reporting the pooled effect of EIT-guided PEEP versus conventional PEEP on static respiratory compliance, stratified by study design (RCT vs observational).

EIT-guided PEEP was associated with a significant improvement in respiratory system compliance compared with conventional PEEP strategies. (MD + 6.81 mL/cm H_2_O, 95% CI 3.73–9.89), with low heterogeneity (I² = 0 %). The effect was more pronounced in observational studies than in RCTs, although the test for subgroup differences was statistically significant, indicating potential differences in patient selection or titration protocols (Fig. 3, Panel B). No indications of publication bias are observed (Fig. 3, Panel A). Asymmetry was not observed on visual inspection and GRADE certainty of evidence was rated as moderate (Table 2).

### Secondary outcomes

#### Driving pressure

**Fig. 4:**
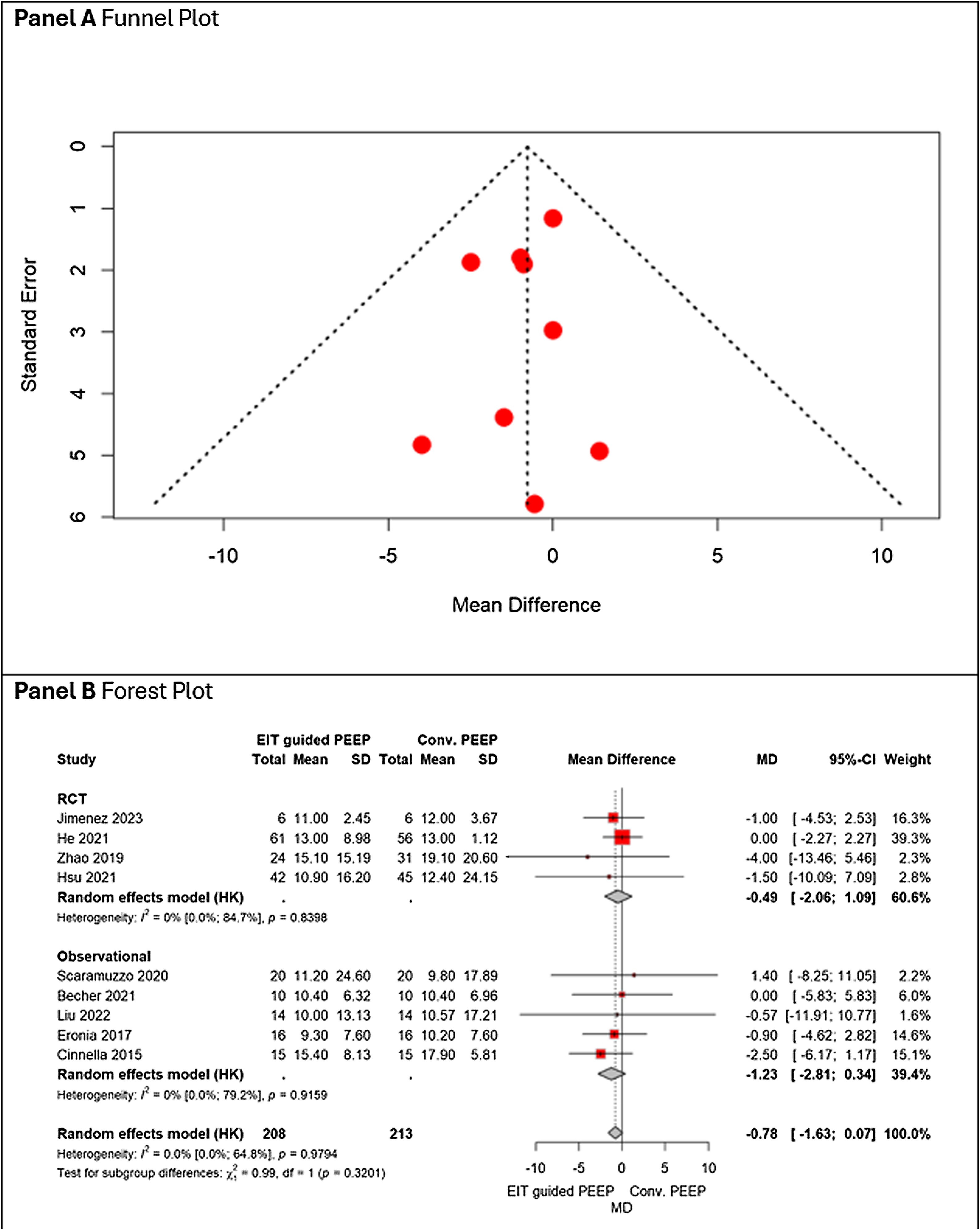
Panel A shows the funnel plot, and Panel B the forest plot, comparing driving pressure between EIT-guided PEEP and conventional PEEP across randomized and observational studies.

No statistically significant difference in driving pressure was observed between EIT-guided PEEP and conventional strategies (MD − 0.78 cm H_2_O, 95% CI − 1.63 to 0.07), with negligible heterogeneity. Results were consistent across RCTs and observational studies (Fig. 4, Panel B). Fig. 4, Panel A, shows that there is no asymmetry on visual inspection and GRADE certainty of evidence was rated as moderate (Table 2).

#### Mechanical power

**Fig. 5:**
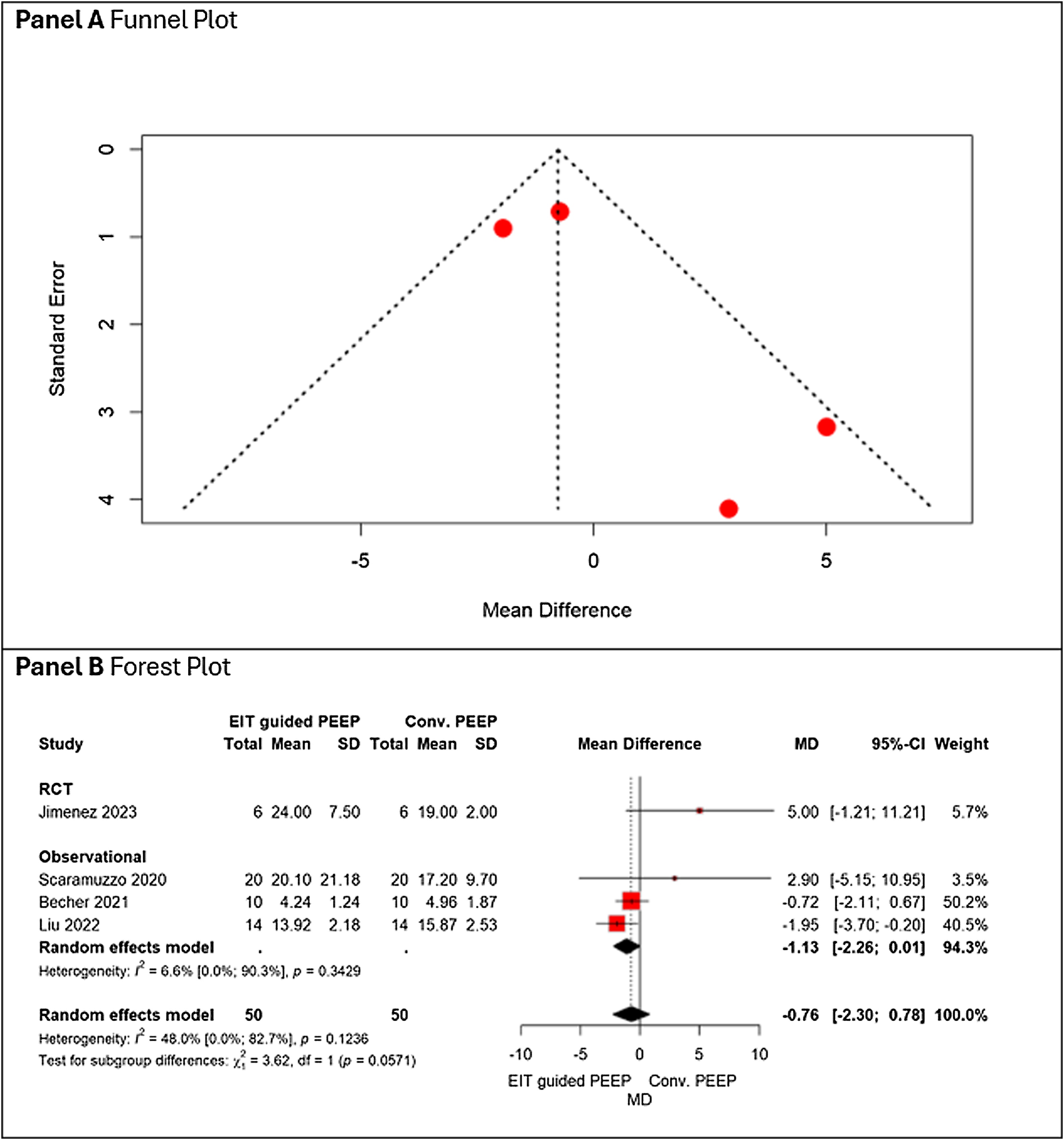
Panel A shows the funnel plot, and Panel B the forest plot, reporting the pooled mean differences in mechanical power between EIT-guided PEEP and conventional PEEP, stratified by study design.

In 4 studies reporting the complete data on MP, EIT-guided PEEP was not associated with a variation in mechanical power compared with conventional approaches (mean difference [MD] −0.76 J/min, 95% CI − 2.30 to 0.78), with heterogeneity (I^2^ = 48%). Subgroup analysis showed no significant differences between randomized controlled trials (RCTs) and observational studies (p = 0.057), (Fig. 5, Panel B). Fig. 5, Panel A, does not reveal any signs of publication bias. No evident asymmetry was observed on visual inspection and GRADE certainty of evidence was rated as low-moderate (Table 2).

#### Mortality

**Fig. 6:**
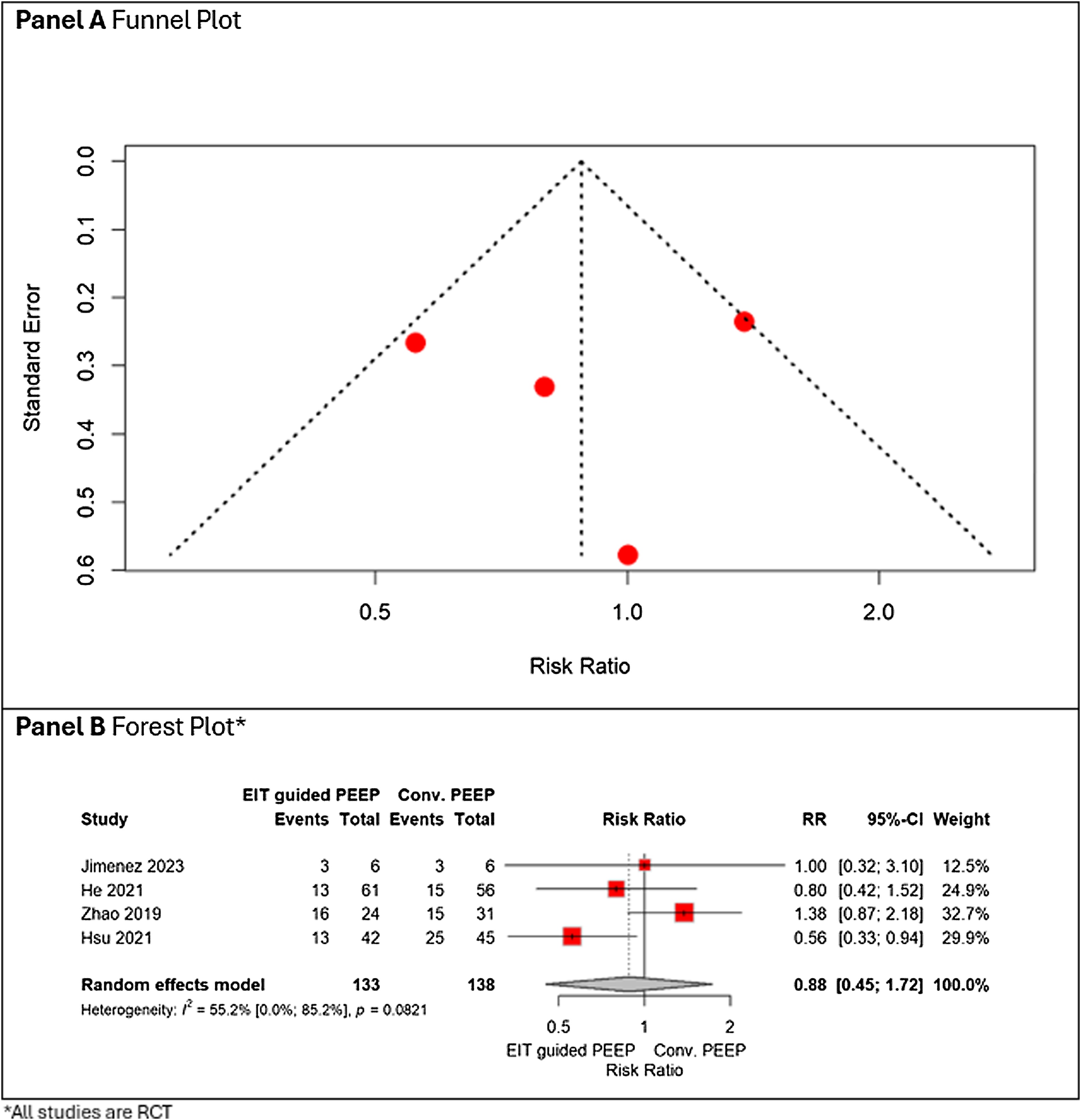
Panel A shows the funnel plot, and Panel B the forest plot, summarizing the risk ratio for mortality in patients managed with EIT-guided PEEP versus conventional PEEP.

Four RCTs studies reported mortality outcomes. EIT-guided PEEP was associated with a non-significant variation in mortality compared with conventional strategies (risk ratio [RR] 0.88, 95% CI 0.45–1.72), with moderate heterogeneity (I^2^ = 55%) (Fig. 6).

## GRADE

For oxygenation and respiratory system compliance, the certainty of evidence was rated as moderate. Downgrading was applied for indirectness, as these represent short-term physiological surrogate endpoints rather than patient-centered outcomes, and for risk of bias, given the inclusion of non-randomized studies judged at serious risk of confounding. No downgrading was applied for inconsistency, as heterogeneity was low, nor for imprecision.

For driving pressure, certainty was also rated as moderate, primarily due to imprecision, as confidence intervals included the null effect. Additional consideration was given to indirectness because driving pressure is a mechanistic rather than clinical outcome.

For mechanical power, certainty was judged as low to moderate, with downgrading for imprecision due to limited number of studies and for risk of bias related to observational designs.

For mortality, certainty was rated as low, reflecting downgrading for imprecision due to limited events and wide confidence intervals. No downgrading for indirectness was applied, as mortality is a direct patient-centered outcome.

Overall, certainty was most commonly reduced due to indirectness of surrogate physiological outcomes, imprecision in selected endpoints, and inclusion of non-randomized evidence at serious risk of bias (Table 2).

## Discussion

In this meta-analysis, we analyzed the effect of an EIT-based versus a conventional PEEP setting strategy in adult patients affected by ARDS. We found that EIT-guided PEEP titration is associated with significant improvements in oxygenation and static respiratory compliance while global mechanical power and driving pressure were not significantly changed, suggesting a redistribution rather than a global reduction of delivered energy.

Our findings align with prior meta-analyses evaluating EIT-guided PEEP titration, but provide added strength in terms of methodological consistency, patient selection, and outcome diversity. Songsangvorn et al. (2024) [10] included 623 patients and demonstrated improved compliance and reduced mechanical power. Yu et al. (2022) [11] focused on oxygenation and compliance in 222 patients and found improved PaO_2_/FiO_2_ but no significant changes in compliance. Gao et al. (2024) [31] addressed a mixed population, including perioperative patients and those with hypoxemic ARF, with no consistent benefits in oxygenation or mechanics. Sanchez-Piedra et al. (2025) and Sarkar et al. (2024) [32,33] both reported mortality benefits (RR = 0.64 and RR = 0.68, respectively), although their analyses were limited by less robust secondary outcome assessment. Jiang et al. (2024) [34] explored the perioperative setting, showing improved intraoperative compliance and oxygenation, but lacked data on ICU outcomes. Findings therefore were inconsistent, probably because the effect of EIT titration strategy depends also on the specific population considered. For this reason we decided, in this analysis, to focus on non-COVID-19 ARDS patients, which are to date, the main ARDS population in the ICU. COVID-19–associated ARDS has been reported to demonstrate pathophysiological features that may differ from those observed in classical ARDS, including variations in respiratory system compliance, the degree and pattern of vascular involvement, the presence of pulmonary microthrombosis, and alterations in ventilation–perfusion matching [35]. Furthermore, heterogeneous respiratory phenotypes [36]have been described during the COVID-19 pandemic, with differences in lung recruitability and responsiveness to PEEP [37].Such variability may affect EIT-derived measurements, the assessment of regional ventilation distribution, and the observed physiological response to PEEP titration.

We found that EIT determines both an improvement of compliance and oxygenation. Oxygenation improvement is not the main objective when titrating mechanical ventilation, especially when it comes at the cost of reducing lung protection [38]. However, the EIT based strategy showed consistent improvement in oxygenation without detrimental effects on lung mechanics, but, on the contrary, improvement of respiratory system compliance.

Indeed, EIT-guided PEEP titration was associated with a significant increase in respiratory system compliance [39,40]. Driving pressure, defined as the difference between plateau pressure and PEEP (ΔP = Pplat − PEEP), serves as an indicator of tidal strain and dynamic stress imposed on the lungs during ventilation [41] and in our case was not different among treatments. This apparent discordance may be related to the difference in tidal volume, as tidal volume was not kept constant across trials. In 2 studies [27,30] EIT titration strategy was associated to an increase of tidal volume, which could have influenced the observed driving pressure. With similar driving pressures, a higher tidal volume may suggest improved compliance; however, this interpretation remains speculative and should be considered hypothesis-generating rather than confirmatory.

Mechanical power (MP), represents a broader measure of ventilatory energy, encompassing not only driving pressure but also tidal volume, respiratory rate, inspiratory flow, and PEEP [[41], [42], [43]].

Although mechanical power did not differ significantly between groups, the observed directional trend toward lower values in the EIT-guided group could suggest a potential shift in how ventilatory energy is distributed. This interpretation remains inferential, as no direct measurements of regional stress/strain or longitudinal assessments were available to substantiate a true redistribution of energy or improved ventilation homogeneity. Another possible interpretation it is possible that global indices such as mechanical power may lack sensitivity to detect regional mechanical improvements, as has been described for other global respiratory parameters, such as the pressure–volume curve. [44]

Most outcomes were short-term physiological measures assessed immediately after PEEP titration and, while mechanistically informative, may not translate into patient-centered outcomes; moreover, the evidence base remains vulnerable to small-study effects due to limited sample sizes and few randomized trials. Previous studies did not show any difference comparing different PEEP titration technique on ARDS mortality [45] but showed an association between driving pressure and mortality independently from PEEP [46]. Therefore, we are not surprised from the lack of difference in mortality between the two titration techniques. Longitudinal protocols of continuous applications of EIT during ICU stay are missing, and current studies are limited to short-time interventions. This might explain the limited effects of outcome and should be explored by future studies.

A methodological aspect that deserves consideration when interpreting our findings is the heterogeneity of PEEP titration strategies adopted to date across studies, particularly within the EIT-guided arms. While conventional PEEP settings were generally based on standardized approaches such as the ARDSNet PEEP/FiO_2_ table or static pressure–volume curves, EIT-based titration employed a wide spectrum of protocols [47]. These varied from decremental PEEP trials based on global or regional compliance, to thresholds for ΔEELI ≥10%, or the minimization of silent spaces [48] or collapse/overdistension ratios. This heterogeneity reflects the absence of an evidence-based consensus for EIT-guided PEEP titration in ARDS, since today most of studies are based on expert recommendation [49]. This aspect represents both a challenge and an opportunity: on one hand, it complicates the direct comparison across studies; on the other hand, it highlights the adaptability and reproducibility of EIT to multiple physiological endpoints, independently from the interval parameter choose as target. Our analysis shows that EIT-guided PEEP titration leads to a more individualized approach, with PEEP levels adjusted to optimize compliance, reduce silent spaces, or improve ventilation distribution.

From a clinical perspective, EIT-guided PEEP titration offers a noninvasive, bedside strategy to tailor mechanical ventilation [[40], [41], [42], [43]]. Higher benefits from EIT-guided PEEP titration may be expected in patients with greater lung recruitability. Previous study has shown that recruitability is higher in the early phase of ARDS, when a larger proportion of potentially recruitable lung tissue is present [39]. These observations support the hypothesis that EIT-guided strategies may be advantageous in selected subgroups, although this requires confirmation in prospective, stratified trials. Future research should aim to stratify patients by ARDS phenotype or surgical context (e.g., post-cardiac surgery) and evaluate the role of modifiers such as BMI, COPD, or baseline severity, as these factors may influence the physiological and clinical response to PEEP and explore long-term outcomes, implementation strategies and cost-effectiveness.

This meta-analysis has some strengths. The present analysis addresses these gaps by restricting inclusion to non–COVID-19 ARDS to enhance physiological homogeneity, applying a design-aware modeling strategy for crossover and paired studies, performing sensitivity analyses restricted to randomized trials. In addition, we applied a structured GRADE framework to assess outcome-level certainty. These methodological refinements aim to strengthen interpretability and clinical relevance of the current evidence base. The exclusion of patients with COVID-19 related ARDS was justified because this condition exhibits distinct mechanical, pathophysiological, and prognostic characteristics. In particular, COVID-19 ARDS is associated with unique patterns of lung recruitability, vascular involvement, and disease progression. Excluding these patients reduced heterogeneity and minimized potential confounding factors. Consequently, this approach enhanced physiological homogeneity and strengthened the internal validity of the analysis. Our study has also some limitations. First, the included studies varied substantially in their methodologies. These strategies were grouped under the broader category of “EIT-guided PEEP” because, despite methodological differences, they are unified by a shared physiology-driven framework, specifically, the use of real-time regional ventilation data obtained from EIT to individualize PEEP, as opposed to relying on conventional global parameters or standardized PEEP tables. Different PEEP titration protocols were employed, with variable time points for outcome assessment and differing criteria for recruitment maneuvers or plateau pressure targets. Furthermore, the lack of standardization in EIT technology across studies, as differences in hardware, software platforms, image reconstruction methods, and analytical algorithms used to derive ventilation and overdistension/collapse indices may introduce heterogeneity and affect comparability. EIT-guided PEEP titration may be operator-dependent, as variability in image interpretation, expertise, and local protocols could introduce additional heterogeneity and performance bias.

Although oxygenation and respiratory mechanics are physiologically meaningful and mechanistically linked to lung protection, they represent short-term surrogate endpoints rather than direct patient-centered outcomes. Therefore, the certainty of evidence reflects confidence in the physiological effect of EIT-guided PEEP rather than definitive clinical benefit. Subgroup analyses showed a consistent direction of effect in randomized trials, although effect sizes were generally larger in observational studies. This pattern was considered when evaluating risk of bias and potential residual confounding within the GRADE framework.

These findings support the physiological rationale for EIT-guided PEEP optimization, while larger trials evaluating clinical outcomes are warranted. Future studies should aim to standardize EIT-guided protocols and directly compare the performance of different titration methods in well-powered randomized trials. Until such data are available, the variability observed across studies should be considered when interpreting pooled results.

These differences limit the comparability of study results and introduce potential bias in the pooled analysis. Second, most studies were single-center, often excluding patients with comorbidities such as COPD, obesity, or postoperative status. This may reduce the generalizability of the findings to broader clinical practice. Moreover, ARDS severity was inconsistently reported, limiting the ability to assess effect modification by disease severity. Indeed, the absence of grey literature screening may increase the risk of publication bias.

Finally, although PaO_2_/FiO_2_ ratio, Crs, and ΔP were consistently reported, mechanical power and clinical outcomes such as duration of mechanical ventilation, ICU stay, or long-term mortality were poorly documented or unavailable in many studies. A standardized approach to reporting physiological and EIT based studies is warranted to facilitate future analysis and clinical translation of experimental evidence.

## Conclusions

EIT-guided PEEP titration improves oxygenation and respiratory system compliance in patients with ARDS, supporting its role as an individualized and physiologically sound ventilatory strategy. These findings suggest that EIT may serve as a valuable bedside tool for optimizing PEEP and improving short-term physiological parameters in ARDS. Future research should focus on long-term outcomes, cost-effectiveness, and the identification of patient subgroups most likely to benefit from EIT-guided strategies.

## CRediT authorship contribution statement

**Michela Rauseo**: Conceptualization, Methodology, Investigation, Data curation, Formal analysis, Visualization, Writing – original draft, Writing – review & editing; **Danila Azzolina**: Formal analysis, Methodology, Validation, Writing – review & editing; **Gaetano Scaramuzzo**: Writing – review & editing; **Mohd Rashid Khan**: Data curation, Formal analysis; **Paolo Vetuschi**: Supervision; **Francesco Paolo Padovano**: Supervision; **Antonello Discenza**: Resources; **Lucia Di Staso**: Resources; **Lucia Mirabella**: Supervision; **Antonella Cotoia**: Supervision; **Savino Spadaro**: Conceptualization, Methodology, Supervision, Writing – review & editing, Project administration; **Gilda Cinnella**: Supervision, Validation, Writing – review & editing.

## Consent for publications

Not applicable.

## Ethics approval and consent to participate

Not applicable.

## Declaration of Generative AI and AI-assisted technologies in the writing process

The authors used AI-assisted tools (OpenAI’s ChatGPT) only for language refinement. The authors reviewed and approved all edits and are responsible for the final content.

## Funding

This study was supported by European Union – Next Generation EU – PNRR M6C2- Investment 2.1Enhancement and strengthening of biomedical research within the national Health Service “PNRR-MCNT2-2023-12377245 - Advanced bedside lung imaging and respiratory muscle monitoring for respiratory support managing in chronic ill patients with acute respiratory failure: From hospital to home.

## Availability of data and material

Data available upon reasonable request to the corresponding author.

## Declaration of competing interest

The authors declare no competing interests.
