# Supplementary material for: Personalized positive-end expiratory pressure using electrical impedance tomography in ARDS patients: a systematic review and meta-analysis
Source: Ann Intensive Care. 2026 Mar 16;16:100049. doi: 10.1016/j.aicoj.2026.100049 (PMC13010124; doi:10.1016/j.aicoj.2026.100049)
Supplement: Supplementary file 1 [file mmc1.docx]

| **Authors** | **Parameters** | **EIT guided PEEP** | **Conventional PEEP** |
| --- | --- | --- | --- |
| Jimenez | PEEP (cmH2O) | 14 | 16 |
|  | RR (bpm) | 16 | 17 |
|  | Vt | 6.1 ml/kg/PBW | 6 ml/kg/PBW |
| He | PEEP (cmH2O) | 8 | 7 |
|  | RR (bpm) | 18 | 17 |
|  | Vt (ml) | 429 | 426 |
| Zhao | PEEP (cmH2O) | 18 | 14 |
|  | RR (bpm) | NA | NA |
|  | Vt | 6.3 ml/kg/PBW | 6.5 ml/kg/PBW |
| Hsu | PEEP (cmH2O) | 16.2 | 17.4 |
|  | RR (bpm) | 20 | 24 |
|  | Vt (ml) | 370 | 400 |
| Scaramuzzo | PEEP (cmH2O) | 14 | 13 |
|  | RR (bpm) | 20 | 19 |
|  | Vt (ml) | 375 | 365 |
| Becher | PEEP (cmH2O) | 16 | 10 |
|  | RR (bpm) | NA | NA |
|  | Vt (ml) | 603 | 348 |
| Liu | PEEP (cmH2O) | 10.43 | 14 |
|  | RR (bpm) | NA | NA |
|  | Vt (ml) | 426 | 407 |
| Eronia | PEEP (cmH2O) | 13 | 9 |
|  | RR (bpm) | NA | NA |
|  | Vt (ml) | 460 | 450 |
| Cinnella | PEEP (cmH2O) | 15 | 7.3 |
|  | RR (bpm) | 12 | 12 |
|  | Vt | 6.9 ml/kg/PBW | 6.4 ml/kg/PBW |

Data are shown as mean values, extracted from the papers. Respirarory Rates (RR) are not available (NA)
