# Supplementary material for: Personalized positive-end expiratory pressure using electrical impedance tomography in ARDS patients: a systematic review and meta-analysis
Source: Ann Intensive Care. 2026 Mar 16;16:100049. doi: 10.1016/j.aicoj.2026.100049 (PMC13010124; doi:10.1016/j.aicoj.2026.100049)
Supplement: Supplementary file 2 [file mmc2.docx]

*Table S2* RoB 2 (randomized trials) Judgement key: Low risk / Some concerns / High risk

| Study | RoB 2 Domain 1: Randomization process | Domain 2: Deviations from intended interventions | Domain 3: Missing outcome data | Domain 4: Measurement of the outcome | Domain 5: Selection of the reported result | Overall RoB 2 |
| --- | --- | --- | --- | --- | --- | --- |
| Jimenez 2023 (randomized crossover) | **Low** – allocation via **opaque sealed envelopes**, 1:1 sequence assignment. | **Some concerns** – **no washout feasible**; also **post-randomization exclusion** of proned patients may introduce bias related to deviations/implementation. | **Some concerns** – exclusions after randomization (e.g., proning) can functionally behave like missingness not completely at random. | **Low** – primary outcomes are physiologic/ventilator-derived (e.g., mechanical power), objectively measured. | **Some concerns** – protocol/registration is present, but selective reporting cannot be fully excluded from the manuscript alone. | **Some concerns** |
| Scaramuzzo 2020 (randomized crossover order) | **Low** – two titration trials performed in **random order (sealed envelope)**. | **Some concerns** – crossover physiology is sensitive to lung-history effects; they added a **20-min washout at clinical PEEP**, but residual carryover is still plausible. | **Low** – short protocol with prespecified measurements; attrition not highlighted. | **Low** – gas exchange/mechanics/EIT outcomes are objective and collected at prespecified time points. | **Some concerns** – full analysis plan not always transparent in the article text; reporting bias cannot be ruled out. | **Some concerns** |
| He 2021 (parallel RCT) | **Some concerns** – randomized groups are clear, but (from the available extracted text) key implementation details (e.g., concealment/sequence generation method) are not fully explicit. | **Some concerns** – likely open-label titration with potential co-intervention differences (not always fully controllable in ventilation trials), though outcomes are largely objective. | **Some concerns** – analysis set excludes “erroneously randomized” misclassified ARDS patients (post-randomization exclusions). | **Low** – mortality and standard physiology measures reduce assessor-driven measurement bias. | **Some concerns** – selective reporting cannot be excluded without a full protocol comparison. | **Some concerns** |
| Hsu 2021 (parallel RCT) | **Some concerns** – states **simple randomization** but masking/allocation concealment details are limited in the extracted Methods. | **Some concerns** – intervention not easily blinded; co-interventions may differ in practice (e.g., escalation pathways), which can influence longer-term outcomes. | **Low–Some concerns** – no strong signal of missingness, but not always fully described for every endpoint. | **Low** for short-term physiology; **Some concerns** for clinical outcomes potentially influenced by open-label management. | **Some concerns** – selective reporting cannot be excluded without protocol/analysis plan cross-check. | **Some concerns** |

*Table S3 ROBINS-I (non-randomized studies) Judgement key: Low / Moderate / Serious / Critical / No information*

| Study | Confounding | Selection of participants | Classification of interventions | Deviations from intended interventions | Missing data | Measurement of outcomes | Selection of reported result | Overall ROBINS-I |
| --- | --- | --- | --- | --- | --- | --- | --- | --- |
| Zhao 2019 (prospective + historical control) | **Serious** – historical control design (2016 controls vs prospective EIT cohort) makes confounding by calendar time/case mix/ICU practices very plausible. | **Serious** – groups assembled differently (prospective consecutive severe ARDS vs retrospective database controls). | **Low–Moderate** – intervention definitions are clear (EIT-guided vs PV-curve-based routine method). | **Moderate** – ventilation management is protocolized but nonrandomized; co-interventions may differ across eras. | **Moderate** – retrospective data may have incomplete capture vs prospective. | **Low** for physiologic endpoints; **Moderate** for outcomes influenced by care pathways. | **Moderate** – protocol transparency limited for all outcomes/analyses. | **Serious** |
| Becher 2021 (sequential, non-randomized within-subject) | **Serious** – explicitly **sequential order without randomization** (ARDSnet first, then EIT protocol), so time/trend and carryover confounding are likely. | **Moderate** – single-arm feasibility enrollment; selection may favor stable patients able to undergo repeated assessments. | **Low** – clear definition of “before” ARDSnet vs EIT optimization. | **Moderate** – adjustments are protocol-driven but open-label; clinical responses could influence subsequent steps. | **Low–Moderate** – short follow-up; missingness not prominent, but device measures (e.g., CO “where available”) suggest partial data. | **Low** – objective physiology/EIT measures. | **Moderate** | **Serious** |
| Liu 2022 (repeated-measures cohort; ARDSnet then EIT titration) | **Serious** – fixed sequence (ARDSnet measurement then EIT titration) and long titration window increase risk of time/carryover confounding. | **Moderate** – inclusion/exclusion clear, but cohort stratified by COPD/non-COPD; residual differences can confound physiologic responses. | **Low** – ARDSnet table vs EIT protocol clearly defined. | **Moderate** – protocol-driven, but clinical instability rules can truncate titration (informative deviation). | **Moderate** – repeated 30-min steps; higher chance of incomplete measurement sets in unstable patients. | **Low** – objective physiologic measurements. | **Moderate** | **Serious** |
| Eronia 2017 (feasibility; consecutive phases) | **Serious** – nonrandomized phase sequence (ARDSnet baseline → EIT-selected PEEP → EIT+2), with strong potential for time/carryover effects. | **Moderate** – multicenter but feasibility sample; selection toward stable, deeply sedated/paralyzed patients. | **Low** – interventions (PEEPARDSnet vs PEEPEIT) clearly defined and measured. | **Moderate** – protocolized but includes repeated RMs and step adjustments that may be influenced by physiology during the test. | **Low** – short protocol; missingness limited (some waveform recording availability varies). | **Low** – objective physiologic endpoints. | **Moderate** | **Serious** |
| Cinnella 2015 (before-after ARDSnet → OLA) | **Serious** – before/after design (baseline ARDSnet then OLA), no randomization; time/recruitment maneuver effects are inseparable from intervention. | **Moderate** – highly selected early mild ARDS; feasibility/physiology focus. | **Low** – ARDSnet baseline and OLA protocol are well described. | **Moderate** – clinical management during OLA (hemodynamic protocol) standardized, but deviations still possible in unstable physiology. | **Low** – short study window; paired measures. | **Low** – objective physiologic outcomes. | **Moderate** | **Serious** |
