# Supplementary material for: Personalized positive-end expiratory pressure using electrical impedance tomography in ARDS patients: a systematic review and meta-analysis
Source: Ann Intensive Care. 2026 Mar 16;16:100049. doi: 10.1016/j.aicoj.2026.100049 (PMC13010124; doi:10.1016/j.aicoj.2026.100049)
Supplement: Supplementary file 3 [file mmc3.docx]

# Search Strategy – Meta-Analysis on EIT-Guided PEEP

The following search strategies were applied in the systematic review and meta-analysis comparing EIT-guided personalized PEEP to conventional approaches in ARDS patients. The search was limited to articles published from January 2012 to March 2025.

## 1. PubMed Search Strategy

("Electrical Impedance Tomography"[Title/Abstract] OR "EIT"[Title/Abstract]) AND ("ARDS"[Title/Abstract] OR "acute respiratory distress syndrome"[Title/Abstract]) AND ("PEEP"[Title/Abstract] OR "positive end-expiratory pressure"[Title/Abstract]) AND ("personalized"[Title/Abstract] OR "individualized"[Title/Abstract] OR "titration"[Title/Abstract]) AND ("clinical trial"[Publication Type] OR "observational study"[Publication Type] OR "randomized controlled trial"[Publication Type])

Filters applied: Publication date from 2012/01/01 to 2025/03/31, Language: English

## 2. Embase Search Strategy

'electrical impedance tomography'/exp OR 'eit':ab,ti AND ('acute respiratory distress syndrome'/exp OR ards:ab,ti) AND ('positive end expiratory pressure'/exp OR peep:ab,ti) AND (individualized:ab,ti OR personalized:ab,ti OR titration:ab,ti) AND ([article]/lim AND [english]/lim AND [2012-2025]/py)

## 3. Web of Science Search Strategy

*TS=("electrical impedance tomography" OR "EIT")
AND TS=("acute respiratory distress syndrome" OR "ARDS")
AND TS=("positive end-expiratory pressure" OR "PEEP")
AND TS=("personalized" OR "individualized" OR "titration")
Refined by: Document Types=(Article OR Clinical Trial)
Timespan: 2012-2025
Languages: English*

## 4. PRISMA Search Summary

Databases searched: PubMed, Embase, Web of Science

Search terms: Electrical Impedance Tomography, EIT, ARDS, PEEP, personalized, individualized, titration

Search limits: Human studies, English language, publication date from 2012 to March 2025

Study types: Randomized Controlled Trials, Observational Studies, Interventional Trials
