# Supplementary material for: Personalized positive-end expiratory pressure using electrical impedance tomography in ARDS patients: a systematic review and meta-analysis
Source: Ann Intensive Care. 2026 Mar 16;16:100049. doi: 10.1016/j.aicoj.2026.100049 (PMC13010124; doi:10.1016/j.aicoj.2026.100049)
Supplement: Supplementary file 4 [file mmc4.docx]

| **Panel A** Funnel Plot (r=0.25)  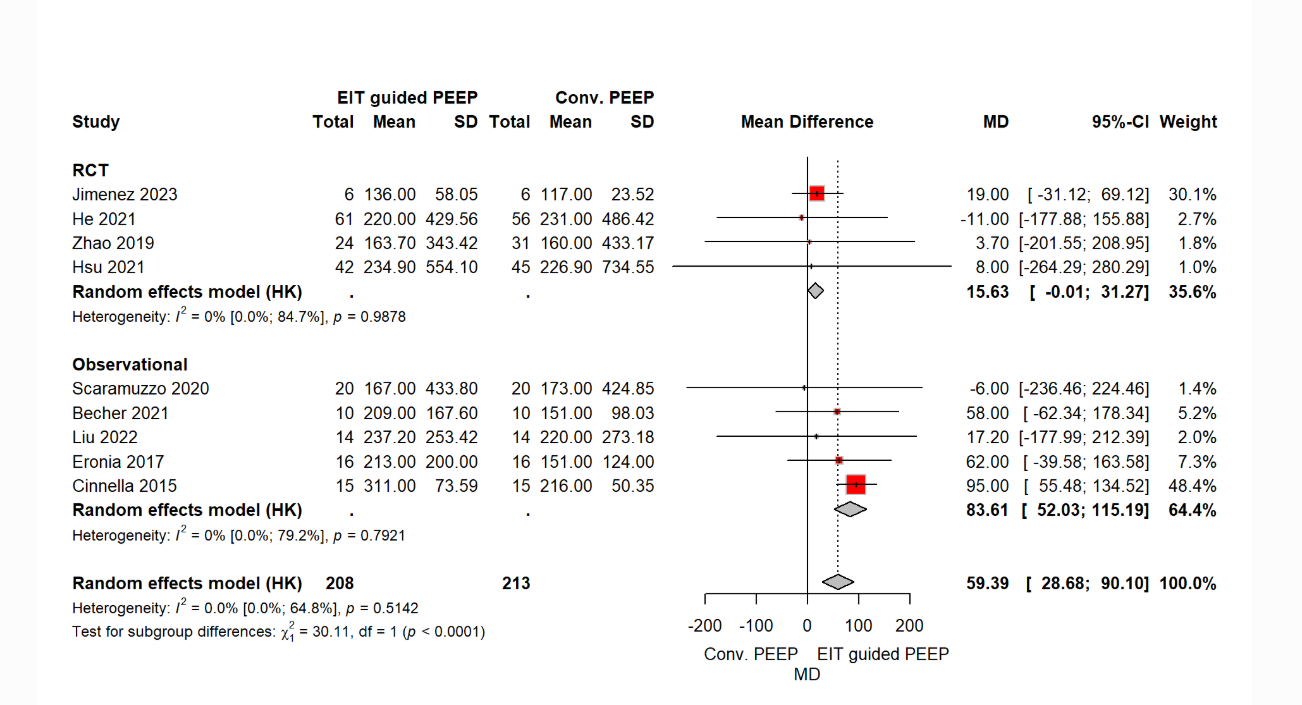 |
| --- |
| **Panel B** Funnel Plot (r=0.75)  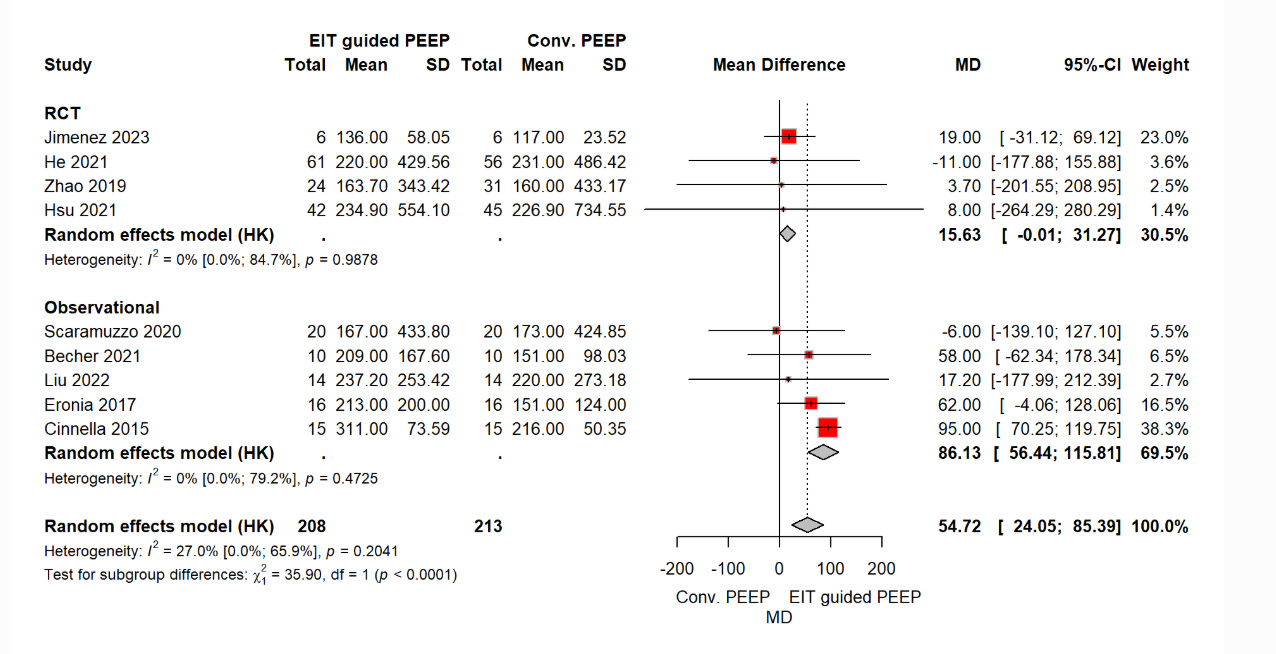 |

**Figure S2:** PaO₂/FiO₂ ratio Meta Analysis. Panel A reports the Funnel plot and Panel B the Forest plot with the pooled estimate and study type (RCT vs Observational) stratification.

| **Panel A** Funnel Plot Funnel Plot (r=0.25)  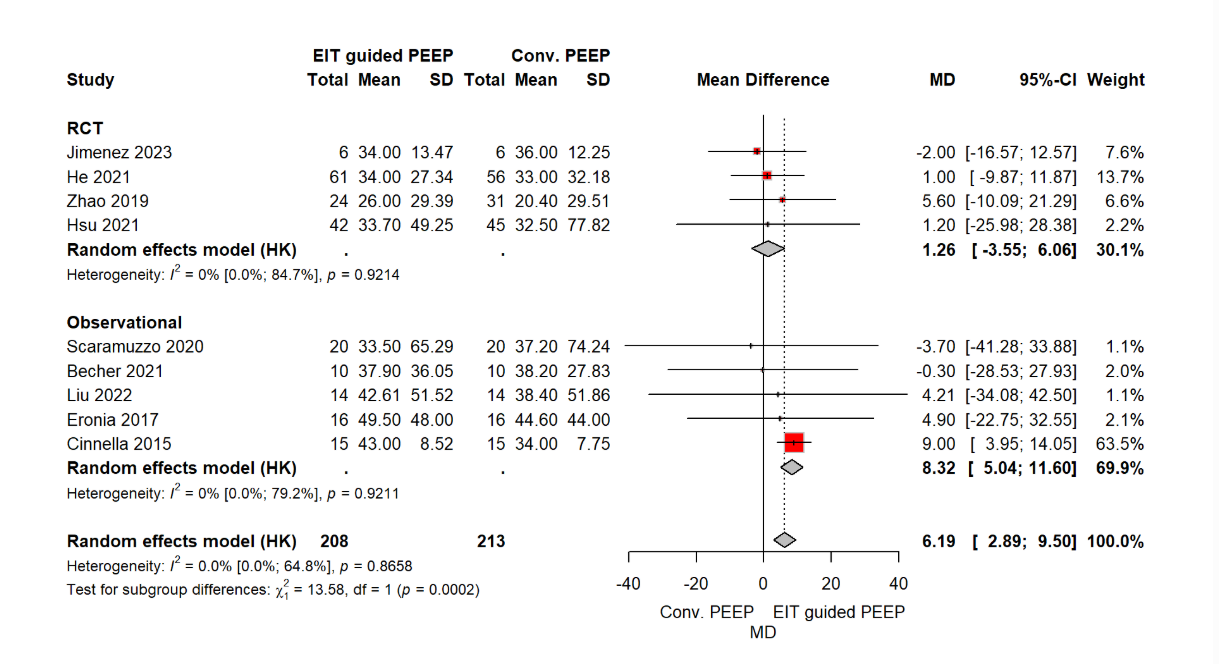 |
| --- |
| **Panel B** Funnel Plot (r=0.75)  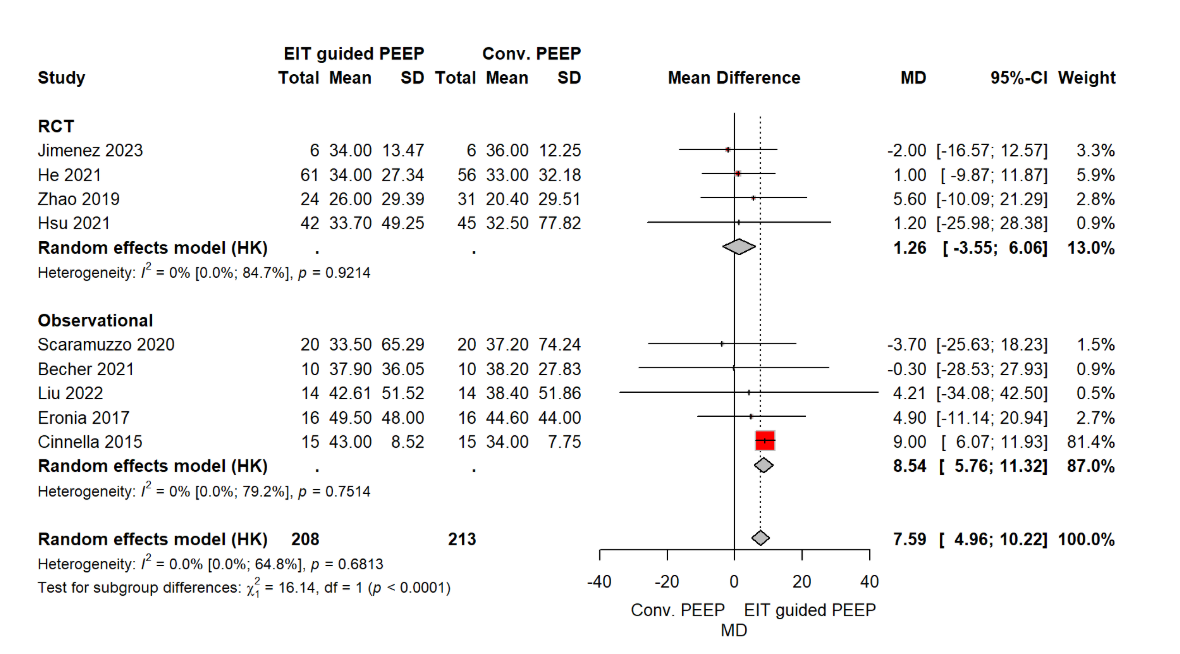 |

**Figure S3:** Respiratory Compliance Meta Analysis. Panel A reports the Funnel plot and Panel B the Forest plot with the pooled estimate and study type (RCT vs Observational) stratification.

| **Panel A** Funnel Plot (r=0.25)  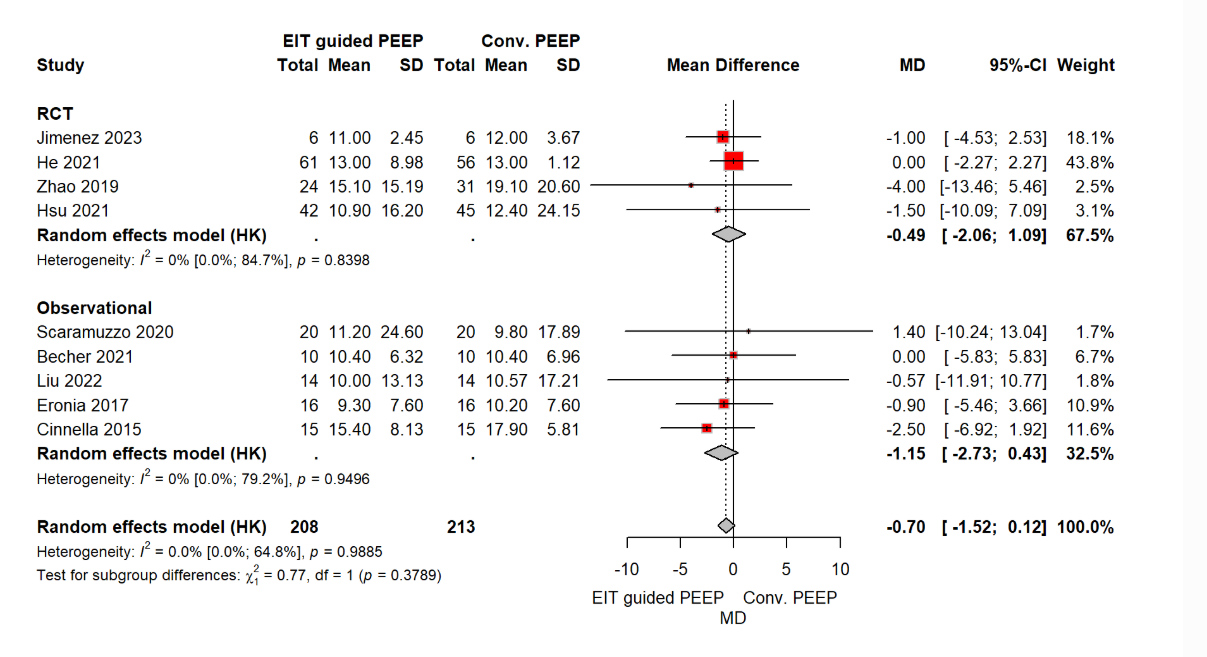 |
| --- |
| **Panel B** Funnel Plot (r=0.75)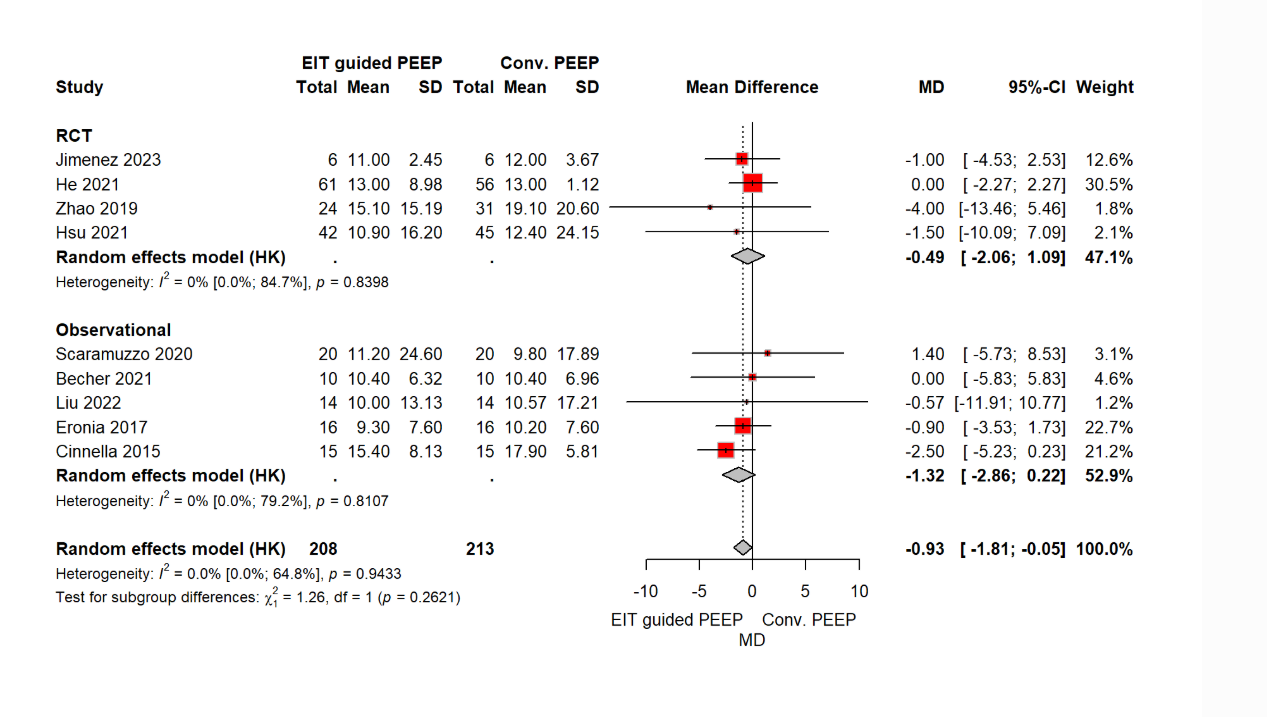 |

**Figure S4:** Driving Pressure Meta Analysis. Panel A reports the Funnel plot and Panel B the Forest plot with the pooled estimate and study type (RCT vs Observational) stratification.

| **Panel A** Funnel Plot  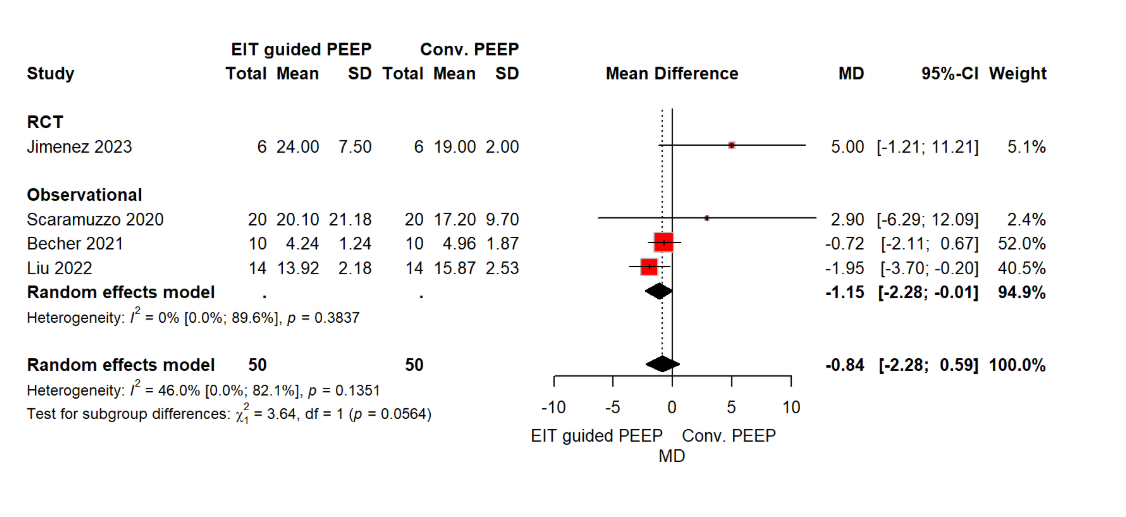 |
| --- |
| **Panel B** Forest Plot  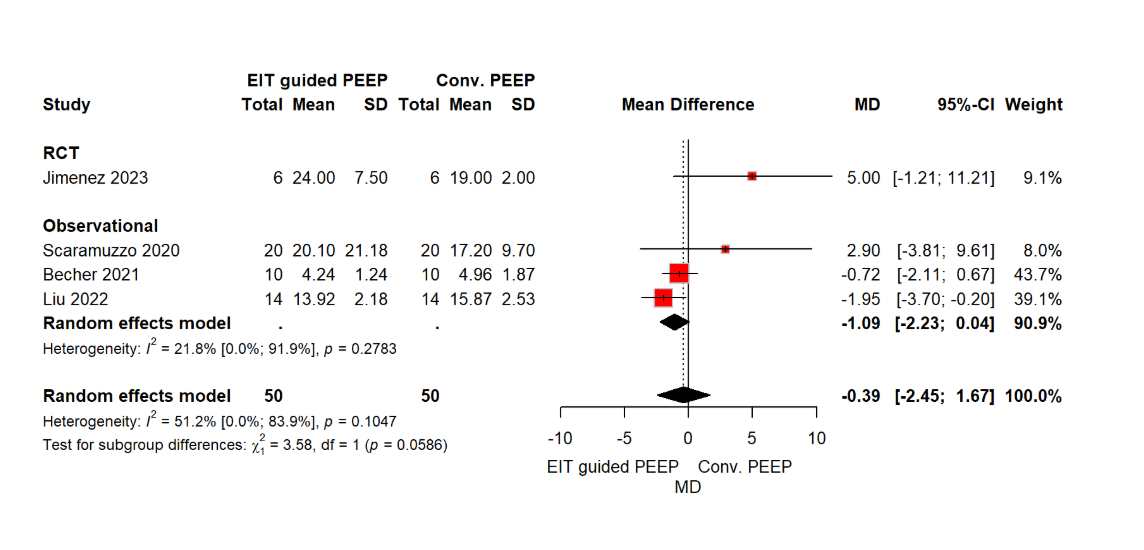 |

**Figure S5:** Mechanical Power Meta Analysis. Panel A reports the Funnel plot and Panel B the Forest plot with the pooled estimate and study type (RCT vs Observational) stratification.
